# Defining Uniform Processes for Remediation, Probation and Termination in Residency Training

**DOI:** 10.5811/westjem.2016.10.31483

**Published:** 2016-11-21

**Authors:** Jessica L. Smith, Monica Lypson, Mark Silverberg, Moshe Weizberg, Tiffany Murano, Michael Lukela, Sally A. Santen

**Affiliations:** * Alpert Medical School of Brown University, Department of Emergency Medicine, Providence, Rhode Island; † University of Michigan, Department of Internal Medicine, Ann Arbor, Michigan; ‡ SUNY Downstate/Kings County Hospital, Department of Emergency Medicine, Brooklyn, New York; § Staten Island University Hospital, Northwell Health, Staten Island, New York; ¶ Columbia University Medical College-NY Presbyterian Hospital, Department of Emergency Medicine, New York, New York; || University of Michigan, Department of Pediatrics, Ann Arbor, Michigan; # University of Michigan, Department of Learning Health Sciences, Ann Arbor, Michigan

## Abstract

It is important that residency programs identify trainees who progress appropriately, as well as identify residents who fail to achieve educational milestones as expected so they may be remediated. The process of remediation varies greatly across training programs, due in part to the lack of standardized definitions for *good standing, remediation, probation, and termination*. The purpose of this educational advancement is to propose a clear remediation framework including definitions, management processes, documentation expectations and appropriate notifications.

*Informal remediation* is initiated when a resident’s performance is deficient in one or more of the outcomes-based milestones established by the Accreditation Council for Graduate Medical Education, but not significant enough to trigger formal remediation. *Formal remediation* occurs when deficiencies are significant enough to warrant formal documentation because informal remediation failed or because issues are substantial. The process includes documentation in the resident’s file and notification of the graduate medical education office; however, the documentation is not disclosed if the resident successfully remediates. *Probation* is initiated when a resident is unsuccessful in meeting the terms of formal remediation or if initial problems are significant enough to warrant immediate probation. The process is similar to formal remediation but also includes documentation extending to the final verification of training and employment letters. *Termination* involves other stakeholders and occurs when a resident is unsuccessful in meeting the terms of probation or if initial problems are significant enough to warrant immediate termination.

## BACKGROUND

Residency training ensures physicians develop the knowledge, skills, and attitudes required to practice medicine independently, and provides the foundation for professional growth.^1^ Recently, the Accreditation Council on Graduate Medical Education (ACGME) and the American Board of Medical Specialties created the Milestones Project to provide competency-based outcomes for trainees. Milestones serve many purposes in both graduate medical education and the accreditation process. Among them, milestones provide transparent expectations, support better longitudinal assessment of trainees, and enhance public accountability through aggregate reporting of competency by specialty.^2^

Residents achieve ACGME milestones at different stages during training.^3^ Some residents require remediation (additional training, assistance or supervision) to meet expectations.^2^, ^4^–^7^ The remediation continuum ranges from residents needing minimal guidance to those who cannot successfully complete training.^8^–^10^ The process of remediation, however, varies greatly across training programs, in part related to the inconsistency in definitions and procedures. Lack of standardized definitions for *good standing, remediation, probation and termination* creates challenges for program directors (PDs) and residents.^11^–^16^

## OBJECTIVES

Establishing shared and consistent definitions of remediation processes will enable training programs to achieve the goals intended by the ACGME, hold the medical profession accountable, and will further engender the public trust.^1^ Although categorizing specific resident deficiencies is beyond the scope of this paper, by incorporating the remediation practices from multiple specialties and identifying the common threads, we provide guidelines for the remediation process independent of the medical specialty.

This paper proposes uniform definitions and processes for *informal remediation, formal remediation, probation and termination.* We examine classification definitions and triggers and elaborate on the documentation and notification requirements. Definition of these four domains were identified through review of the literature. To achieve consensus and external content validity of our model, we conducted semi-structured interviews of seven PDs from different programs and five designated institutional officers (DIOs) or deans of graduate medical education (GME) from five institutions. Interviews began with open-ended questions to allow PDs, DIOs, and the GME office to describe their processes for remediation, including probation and termination. They were then asked to provide definitions for each of the four domains identified within our model. Themes were abstracted and compared with our definitions. While we noted minor variances regarding how programs or institutions applied different aspects of the remediation process, the central definitions and sequence followed were consistent with our model.

## DESIGN

### Informal remediation

Informal remediation represents the first step in the process and is initiated when warning signs of problems exist but problems are not so significant to warrant immediate formal remediation.^17^ This stage serves as a critical opportunity to document the process if the resident fails to improve and there is an ultimate need to escalate the remediation. After surveying various PDs and GME officials in the authors’ own institutions, we found that some programs create official documentation in the resident’s official file; others use e-mail communication with the resident to document the informal remediation conversation; “Confidential Notes” may be created to remain peer-review protected; other PDs use separate “shadow files,” which are disposed of once the resident course corrects over time. It is important to document the resident’s strengths, deficiencies, expectations for improvement, an observation period and progress during remediation. If the resident subsequently requires formal remediation, this initial documentation will serve as the official file.

During informal remediation, the PD, resident and clinical competency committee (CCC) are engaged, but not the GME office (which consists of the DIO and/or the deans of GME). Provided the resident remediates, informal remediation is not disclosed in the final verification of training or employment letters.

### Formal remediation

Formal remediation represents the next step in the process of managing residents with deficiencies. This stage should be implemented when the resident has failed to correct identified deficiencies during informal remediation, or problems are significant enough to warrant immediate formal remediation. The length of formal remediation is determined by the PD, often at the recommendation of the CCC, and should be well defined.

First, the failed informal remediation process and the unresolved deficiencies should be documented to provide evidence that formal remediation is necessary. Next, an updated corrective action plan should be documented with expected outcomes, a time frame for reassessment, and potential consequences if the remediation is not successful. Program and/or institutional grievance and due process policies should be made available to the resident. The PD should provide the resident a formal letter to be signed by both parties to acknowledge receipt and understanding. This documentation should be maintained in the resident’s permanent file. The GME office should be notified that the resident has been placed on formal remediation. Some GME offices may want to review and contribute to the formal remediation letter or plan. In most cases, provided the resident successfully remediates the deficiency, formal remediation documentation is not disclosed in the resident’s final verification of training or employment letters.

### Probation

Probation is initiated when a resident fails to correct deficiencies during formal remediation or if problems are significant enough to warrant immediate probation. Some programs prescribe a maximum of six months of formal remediation, after which the resident is placed on probation if identified deficiencies are not corrected. Further, if resident difficulties require extension of training, the resident may need to be placed on probation, depending on institutional guidelines. The time period for probation should be concrete and follow due process if there is consideration of non-renewal of contract or termination.

The process during probation is similar to formal remediation. The PD should place formal documentation into the resident’s file noting the status, expected outcomes, revised remediation plan, a time frame and consequences if the remediation during probation is not successful. Probation may include limitations on clinical responsibilities. Both the PD and the trainee sign the documentation to ensure receipt and understanding. Institutional grievance policies and due process policies must be given to the resident. The resident’s training responsibilities may need to be modified.

The GME office must be involved in resident probation. In addition, the CCC, department chair, and faculty participating in the resident’s remediation should collaborate. Based on GME office guidance, the institution’s legal counsel might be involved to ensure due process. Probation is disclosed in the final verification of training, employment letters and letters of reference.

If the resident fails to meet the requirements of probation, the program may choose to not renew the employment contract or to terminate the resident. The resident on probation should be informed that the contract will not be renewed for the following academic year, and PDs have the ability to rescind the non-renewal process should the resident demonstrate significant progress. Alternatively, the program may proceed with termination.

### Termination

Termination occurs when a resident fails to meet the terms of probation or if initial problems are significant enough to warrant immediate termination. It is important to document how the resident failed to resolve the identified deficiencies during remediation and probation. The GME office, legal counsel and human resources are often involved in termination. If there is a house officer union, a representative may need to be involved. Termination disclosure is included in the final verification of training, employment letters, and in letters of reference.

## IMPACT

There is significant variation among programs regarding definitions and processes of remediation, probation, and termination.^16^ We provide a consensus framework for defined triggers, associated documentation, and disclosure practices. If remediation is not adequately documented and a clear process is not followed, this can hamper and affect the outcomes of formal grievance processes; thus, this schema has a standardized component to avoid that pitfall.

PDs are responsible for resident remediation and may be bound by requirements from the DIO, the GME office, the department, human resources, legal counsel or unions. It is important for PDs to work closely with key stakeholders, reach out early in the remediation process, and be aware of local policies. Although we provide clear lines of distinction at each remediation stage, the lines sometimes blur. This occurs from insufficient documentation, lack of transparency, and poor communication. Therefore, creating clarity through good documentation and open communication is critical.

This is an initial model to help clarify the definitions in the remediation process. Our remediation schema (Figure) will prove a valuable reference for PDs to provide clear instructions on how to navigate remediation and the documentation and disclosures that are required. This will help communication between residents and faculty, so trainees are aware of the process and consequences if their performance requires remediation. Ultimately, every program must ensure that they have well-defined guidelines to deal with issues of remediation, probation and termination. Next steps might be to collect further validity evidence and utility for the model.

## Figures and Tables

**Figure:**
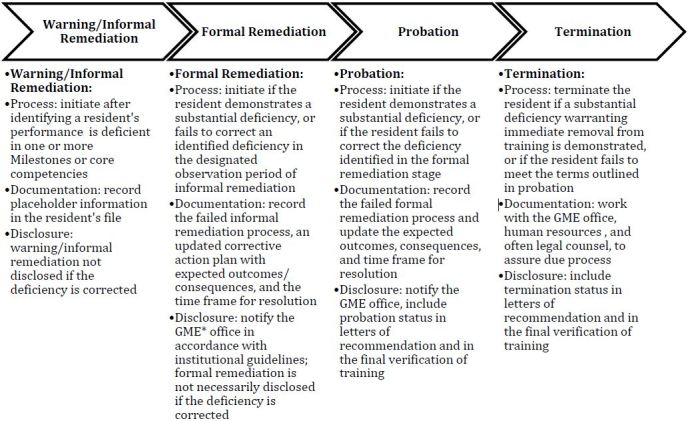
Remediation schema for residents at risk of not meeting educational milestones during their training. ^*^GME, graduate medical education
